# Intrafractional vaginal dilation in anal cancer patients undergoing pelvic radiotherapy (DILANA) – a prospective, randomized, 2-armed phase-II-trial

**DOI:** 10.1186/s12885-020-6547-7

**Published:** 2020-01-21

**Authors:** Nathalie Arians, Matthias Häfner, Johannes Krisam, Kristin Lang, Antje Wark, Stefan A. Koerber, Adriane Hommertgen, Jürgen Debus

**Affiliations:** 1 Department of Radiation Oncology, Heidelberg University Hospital, Im Neuenheimer Feld 400, D-69120 Heidelberg, Germany; 2 Heidelberg Institute of Radiation Oncology (HIRO), Heidelberg, Germany; 3 National Center for Tumor diseases (NCT), Heidelberg, Germany; 4 Institute of Medical Biometry and Informatics, Heidelberg University, Heidelberg, Germany; 5 Clinical Cooperation Unit Radiation Oncology, German Cancer Research Center (DKFZ), Heidelberg, Germany; 6 Heidelberg Ion-Beam Therapy Center (HIT), Department of Radiation Oncology, Heidelberg University Hospital, Heidelberg, Germany; 7 German Cancer Consortium (DKTK), partner site Heidelberg, Heidelberg, Germany

## Abstract

**Background:**

The incidence of anal cancer is rising in the last decades and more women are affected than men. The prognosis after chemoradiation is very good with complete remission rates of 80–90%. Thus, reducing therapy-related toxicities and improving quality of life are of high importance. With the development of new radiotherapy techniques like IMRT (Intensity-modulated radiotherapy), the incidence of acute and chronic gastrointestinal toxicities has already been reduced. However, especially in female anal cancer patients genital toxicities like vaginal fibrosis and stenosis are of great relevance, too. Up to now, there are no prospective data reporting incidence rates, techniques of prevention or impact on quality of life. The aim of the DILANA trial is to evaluate the incidence and grade of vaginal fibrosis, to optimize radiotherapy by reducing dose to the vaginal wall to minimize genital toxicities and improve quality of life of anal cancer patients.

**Methods:**

The study is designed as a prospective, randomized, two-armed, open, single-center phase-II-trial. Sixty patients will be randomized into one of two arms, which differ only in the diameter of a tampon used during treatment. All patients will receive standard (chemo) radiation with a total dose of 45–50.4 Gy to the pelvic and inguinal nodes with a boost to the anal canal up to 54–60 Gy. The primary objective is the assessment of the incidence and grade of vaginal fibrosis 12 months after (chemo) radiation depending on the extent of vaginal dilation. Secondary endpoints are toxicities according to the CTC AE version 5.0 criteria, assessment of clinical feasibility of daily use of a tampon, assessment of compliance for the use of a vaginal dilator and quality of life.

**Discussion:**

Prospective studies are needed evaluating the incidence and grade of vaginal fibrosis after (chemo) radiation in female anal cancer patients. Furthermore, the assessment of techniques to reduce the incidence of vaginal fibrosis like intrafractional vaginal dilation as well as other radiotherapy-independent methods like using a vaginal dilator are essential. Additionally, implementation of a systematic assessment of vaginal stenosis is necessary to grant reproducibility and comparability of future data.

**Trial registration:**

The trial is registered with clinicaltrials.gov (NCT04094454, 19.09.2019).

## Background and rationale

With an incidence of 1/10000, anal cancer accounts for 1–2% of all gastrointestinal tumors and 2–4% of all colo−/anorectal cancers [1]. Incidence is increasing in the last decades and women are proportionally more often affected than men [1]. Apart from very early tumor stages, standard therapy consists of primary chemoradiation according to national and international guidelines [1–5]. This therapy proofed to be the most effective therapy with the chance of sphincter preservation and thus preservation of continence. Chances of curation, especially in early stage disease, are very good with rates of complete tumor remission of about 80–90% [1]. In general, therapy-associated toxicity is the limitating factor for primary chemoradiation. Regarding the good prognosis of patients with anal cancer, reduction of acute and especially chronic toxicities is an important step to warrant a good quality of life. In the last decades, many efforts have been made to improve radiotherapy techniques to increase tumor control and decrease toxicities. New technical developments in the field of radiotherapy like IMRT (intensity-modulated radiotherapy), including VMAT (volumetric arc therapy) and Tomotherapy have resulted in an improved sparing of organs at risk (OARs) like rectum, bowel and bladder leading to reduced toxicity of pelvic radiotherapy. However, the focus has mainly been on gastrointestinal toxicities [6–11]. Additionally, the incorporation of FDG-PET into treatment planning offers the opportunity for sparing of functional bone marrow as another organ at risk, thus reducing hematological toxicity [12]. There are only very few data on genital toxicities like vaginal fibrosis and stenosis [13–15]. As mostly women are affected, this is a relevant topic. Genital toxicities of radiotherapy like vaginal fibrosis are mostly reported from women receiving radiotherapy for cervical or endometrial cancer [16–25]. Due to the anatomical proximity of anal canal and vagina, vaginal fibrosis is also a relevant side effect of radiotherapy for anal cancer, which has been widely underestimated until a few years ago. There are only few and inconsistent retrospective data reporting rates of vaginal fibrosis after radiation treatment of female anal cancer patients of 1.6–80% [26, 27]. Furthermore, there is no established method to assess vaginal fibrosis, making it difficult to compare data and leading to the inconsistent data reported. Additionally, as a lack of prospective data, no clear recommendations for prophylaxis and therapy of vaginal fibrosis do exist. Current recommendations differ by center and are extrapolated from recommendations for women treated with radiotherapy for gynecological cancers. For these patients recommendations for the regular use of a vaginal dilator after finishing radiotherapy exist to prevent from vaginal stenosis (International Clinical Guideline Group, National Forum of Gynaecological Oncology Nurses, UK. International Guidelines on Vaginal Dilation After Pelvic Radiotherapy. Oxon: Owen Mumford; 2012).

As a result of the close topographic relationship of the anal canal and the vagina, the dorsal as well as the ventral wall of the collapsed vagina are often included in the radiation field, thus receiving high radiation doses. As we know that there is a dose-relationship for the incidence of most toxicities, reducing the dose to at least some parts of the vagina could reduce vaginal fibrosis [28, 29]. A dosimetric analysis could already show an advantage of vaginal dilation regarding radiation dose at the vaginal wall [30]. A further clinical trial with 10 patients was also able to show a reduction of the median total dose on the vaginal wall by using vaginal dilators during radiotherapy [28]. Furthermore, we already know from other hollow organs like the rectum, that sparing of some parts of the circumference results in lower toxicity rates. That’s why some institutions already developed strategies to at least spare some parts of the vaginal wall circumference. For this purpose, vaginal dilation using commercially available tampons is often applied during irradiation. But there are no prospective clinical data showing a positive effect of vaginal dilation on the rate of vaginal fibrosis or giving any details on the extent of vaginal dilation needed to achieve a positive effect.

The aim of this prospective, randomized study is to evaluate the incidence and grade of vaginal fibrosis in female anal cancer patients treated with (chemo) radiotherapy depending on the extent of vaginal dilation. For this purpose, we aim to establish a standardized system for the assessment of vaginal fibrosis, to grant reproducibility and comparability of future data. The greater aim is to optimize radiotherapy of anal cancer patients by reducing dose to the vaginal wall to reduce genital toxicities and improve quality of life.

## Methods/Design

### Study design

The study is designed as a prospective, randomized, two-armed, open, single-center phase-II-trial evaluating the incidence and extent of vaginal fibrosis in female anal cancer patients treated with (chemo) radiotherapy. We aim to evaluate if an increased intrafractional vaginal dilation using a special tampon is associated with a lower incidence and/or grade of vaginal fibrosis. After obtaining written informed consent, patients fulfilling the inclusion criteria will be randomized into one of the two arms, which differ only in the kind of tampon used during treatment. All patients will receive standard (chemo) radiotherapy to the pelvic and inguinal (if required) nodes with a total dose of 45–50.4 Gy (single dose 1.8–2 Gy) and a sequential or integrated boost to the anal canal up to 54–60 Gy. All patients will be advised to use a vaginal dilator regularly starting 6–8 weeks after finishing radiotherapy to prevent from vaginal stenosis.

### Study objectives

Primary endpoint is the incidence of vaginal fibroses >/= Grade 1 depending on the extent of vaginal dilation measured 12 months after radiotherapy. A commercially available vaginal dilator set will be used as measuring device. The grading of vaginal stenosis will be determined as difference of the diameter of vaginal dilator to the baseline. A reduction of the diameter of < 20% is defined as vaginal stenosis Grade 1, a reduction of 20–35% as Grade 2, a reduction of > 35–49% as Grade 3 and a reduction >/=50% as Grade 4 (Table 1).

**Table 1:** Assessment of vaginal stenosis using a commercially available vaginal dilator set

		Baseline				
Follow-up	Diameter	35 mm	30 mm	25 mm	20 mm	15 mm
	35 mm	0				
	30 mm	I°	0			
	25 mm	II°	I°	0		
	20 mm	III°	II°	II°	0	
	15 mm	IV°	IV°	III°	II°	0

Secondary endpoints are clinical symptoms and acute and chronic toxicities according to the CTC AE version 5.0 criteria, assessment of clinical feasibility of daily use of a tampon for vaginal dilation, assessment of the compliance for the use of a vaginal dilator and quality of life assessed with the EORTC-QLQ30/−ANL27 questionnaires.

### Sample size calculation

We hypothesize that the rate of vaginal stenosis Grade 1 or higher 12 months after radiotherapy is lower in the experimental group using extended vaginal dilation during radiotherapy as compared to the control group. Rates of vaginal stenosis of 50% have been observed in previous patient collectives and we hypothesize that a reduction to 25% is possible in the experimental group. The null hypothesis (H_0_: π_C_ ≤ π_E_; „the rate of vaginal stenosis using a normal tampon is lower or equal to the rate of stenosis using a “special tampon”) will be tested at the one-sided significance level of α = 0.15. Using the Chi^2^-test, a sample size of 52 patients (26 per arm) is necessary to achieve a power of 1-β = 0.80 for the alternative hypothesis assuming a rate of vaginal stenosis in the experimental group of π_E_ =25% and of π_C_ = 50% in the control group). Assuming a drop-out-rate of 12.5%, 60 patients will be included in the study. A logistic regression model will be used, stratifying for the use of simultaneous chemotherapy (yes/no), thus expecting an additional increase in power. Calculations were performed using ADDPLAN, Version 6.1.

### Statistical analysis

The primary analysis includes all enrolled patients (Intent-To-Treat-Population (ITT)). In addition, a per-protocol analysis will be performed. The primary endpoint “vaginal stenosis Grade 1 or higher 12 months after radiotherapy (yes/no)” will be assessed using a logistic regression model adjusting for the factor simultaneous chemotherapy (yes/no), applying a one-sided significance level of α = 0.15. Using this relatively liberal significance level reflects the phase-II character of the trial, and results in a sample size which can be enrolled in a realistic timeframe, yielding an adequately high power. The associated odds ratio will be determined together with a two-sided 70%-confidence interval. Missing values for the primary outcome will be imputed using multiple imputation [31]. Methods of descriptive data analysis will be used to evaluate the secondary endpoints and safety data. This includes calculation of appropriate measures of the empirical distribution and graphical display of the results. Details of the analysis will be specified in a statistical analysis plan which will be finalized before database lock. All analyses will be done using SAS version 9.4 or higher.

### Participants/patient selection

Inclusion criteria according to the protocol are:

- Female patient
- Histologically confirmed squamous anal cancer
- Indication for definitive or postoperative radiotherapy*
- ECOG 0–2
- Age > 18 years
- Written informed consent

Exclusion criteria are the following:

- patient’s refusal or incapability of informed consent
- no vaginal dilation possible prior to radiation treatment start
- clinical evidence of tumor infiltration of the vagina or vulva
- prior pelvic irradiation (if direct field border or even overlap of radiation fields assumed)
- participation in another clinical trial which might influence the results of the DILANA trial
- pregnancy/nursing period or inadequate contraception in women with child bearing potential

Simultaneous chemotherapy is NOT an exclusion criterion.

*indications for chemoradiotherapy are in detail: patients staged cT2-cT4 cN0 cM0 or showing positive lymph nodes (N+) or tumors with poor differentiation (G3) or tumors with affection of the dentate line or even the anal sphincter or cases of R1/2 resection (e.g. in case of excision of an “accidental” tumor under the assumption of a benign disorder like anal tag or hemorrhoids).

### Investigation schedule (Fig. 1)

The oncological treatment concept for each patient is based on interdisciplinary assessment following approved standard therapies and guidelines.

**Fig. 1:**
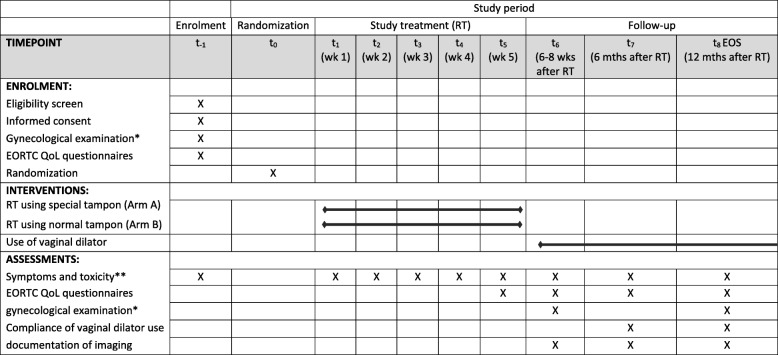
Study schedule

After screening including gynecological examination and obtaining written informed consent patients will be randomly assigned to one of the two study arms using a validated online tool. Patients in arm A will use a special tampon with extended vaginal dilation (diameter 28 mm), patients in Arm B will use a normal commercially available tampon (diameter 12-13 mm) during radiotherapy. As part of the gynecological examination, measuring the vaginal diameter using a vaginal dilator set will be performed which will serve as baseline measurement. As part of the screening, clinical symptoms according to the CTC AE v5.0 criteria and quality-of-life assessed with the EORTC-QLQ30/−ANL27 questionnaires will be evaluated.

#### Radiotherapy-planning

All patients will receive a CT scan for treatment planning with one of the tampons described above, depending on the treatment arm. 3-dimensional radiotherapy planning using a clinically authorized treatment planning system will be performed. All patients will be treated with an image-guided, conformal radiotherapy technique (IMRT). For treatment planning and dose optimization the outer contour of the following organs at risk will be contoured:

- Bladder: Whole organ including the bladder neck.
- Rectum: From the ano-rectal sphincter to the recto-sigmoid junction.
- Sigmoid: From the recto-sigmoid junction to the left iliac fossa.
- Bowel: Outer contour of bowel loops including the mesenterium.
- Femoral heads: Both femoral head and neck to the level of the trochanter minor.
- Vagina: whole vagina from the introitus to the cervix including the tampon and the surrounding soft tissue of the vaginal wall. The ventral and posterior half of the vaginal wall are contoured separately. A possible overlap of the PTV with the vagina (PTV_Vagina) will be documented separately. The anatomical vaginal reference points defined at the level of the Posterior-Inferior Border of Symphysis (PIBS) and ± 2 cm will be applied.
- Cauda equina: dural sac from the second lumbar vertebra to the sacrum.

Dose constraints for organs at risk are according to the Quantec data (Table 2). In case of overlap between the PTV and the Vagina, no underdosage in the PTV will be tolerated.

**Table 2:** Dose constraints for organs at risk

Range	Organ at risk	Parameter	Constraint Optimal (tolerable)
1	Bladder	D_mean_	< 30 Gy (< 40 Gy)
2	Sigma	D_max_	< 50 Gy (< 57 Gy)
3	Colon	D_max_ 200 cc	< 50 Gy (< 54 Gy) < 30 Gy
4	Cauda equina	D_max_	< 25 Gy (< 45 Gy)
5	Femoral heads	D_mean_	< 30 Gy (< 35 Gy)
6	Vagina	D_mean_	< 40 Gy

*Gy* Gray, *D* Dose

A total dose of 45–50.4 Gy (single dose 1.8–2 Gy) to the pelvic and inguinal (if required) lymphatic drainage with a sequential or integrated boost to the anal canal up to 54–60 Gy (single doses 1.8–2.2 Gy) will be applied.

#### Target volume definition

Gross Tumor Volume (GTV).

GTV_PT: macroscopic primary tumor (on MRI/CT)

- GTV_LN: macroscopic lymph node metastases (short axis diameter > 1 cm [exept inguinal] and/or other morphological imaging signs of malignancy, ultrasound correlation can be used if necessary)

Clinical Target Volume (CTV) according to Ng et al. [32]

- CTV_BoostPT:
  - if macroscopic primary tumor: GTVPT + 5–10 mm, complete anal canal, sphincter muscle
  - in case of Rx/R1-situation: preoperative tumor extension + 5–10 mm, complete anal canal, sphincter muscle
- CTV_BoostLN: GTVLN + 3 mm
- CTV_LAD (lymphatic drainage):
  - peri−/mesorectal, presacral, internal and extern iliacal, inguinal (may not be necessary in case of T1 cN0), ischiorectal fossa, perineal
  - cranial border: promontory

Planning Target Volume (PTV):

- PTV_BoostPT: CTV_BoostPT + 5–10 mm
- PTV_BoostLN: CTV_BoostLN + 5–10 mm
- PTV_LAD: CTV_LAD + 5–10 mm

#### Monitoring during treatment/adverse events

Patients are evaluated weekly during radiotherapy. Radiotherapy-related toxicities are assessed using the National Cancer Institute (NCI) Common Toxicity Criteria (CTC) version 5.0 (Table 3). Toxicity will be evaluated pre-treatment, weekly during radiation therapy and at follow-up. Expectable possible acute toxicities (up to 3 months post radiation therapy) are fatigue, loss of appetite, weight loss, skin toxicity, nausea, vomiting, irritable bowel syndrome, diarrhea, proctitis, dysuria, hematological toxicity, vaginal dryness, vaginal discharge and vaginal inflammation. All acute toxicities should resolve within a few weeks after radiation therapy. Late side effects are rare and are defined as symptoms appearing at least 3 months post radiation. These could include chronic diarrhea, malabsorptive syndrome, lymphedema, chronic bladder inflammation, enterocolitis, strictures, fibroses, ulcers, chronic bleeding, vaginal dryness, vaginal discharge and vaginal fibrosis/stenosis. Very rare symptoms are sphincter insufficiency with fecal incontinence, fistulation, perforation, peritonitis, intestinal necrosis or ileus necessitating surgical intervention.

**Table 3:** Toxicities assessed during and after radiotherapy according to the CTC AE v5.0 criteria

Symptom	1°	2°	3°	4°	5°
Proctitis – A disorder characterized by inflammation of the rectum.	Rectal discomfort, intervention not indicated	Symptomatic (e.g., rectal discomfort, passing blood or mucus); medical intervention indicated; limiting instrumental ADL	Severe symptoms; fecal urgency or stool incontinence; limiting self care ADL	Life-threatening consequences; urgent intervention indicated	Death
Diarrhea – A disorder characterized by an increase in frequency and/or loose or watery bowel movements.	Increase of <4 stools per day over baseline; mild increase in ostomy output compared to baseline	Increase of 4–6 stools per day over baseline; moderate increase in ostomy output compared to baseline; limiting instrumental ADL	Increase of > = 7 stools per day over baseline; hospitalization indicated; severe increase in ostomy output compared to baseline; limiting self care ADL	Life-threatening consequences; urgent intervention indicated	Death
Cystitis noninfective – A disorder characterized by inflammation of the bladder which is not caused by an infection of the urinary tract	Microscopic hematuria; minimal increase in frequency, urgency, dysuria, or nocturia; new onset of incontinence	Moderate hematuria; moderate increase in frequency, urgency, dysuria, nocturia or incontinence; urinary catheter placement or bladder irrigation indicated; limiting instrumental ADL	Gross hematuria; transfusion, IV medications, or hospitalization indicated; elective invasive intervention indicated	Life-threatening consequences; urgent invasive intervention indicated	Death
Anal mucositis – A disorder characterized by ulceration or inflammation of the mucous membrane of the anus	Asymptomatic or mild symptoms; intervention not indicated	Symptomatic; medical intervention indicated; limiting instrumental ADL	Severe symptoms; limiting self care ADL	–	–
Vaginal dryness – A disorder characterized by an uncomfortable feeling of itching and burning in the vagina	Mild vaginal dryness not interfering with sexual function	Moderate vaginal dryness interfering with sexual function or causing frequent discomfort	Severe vaginal dryness resulting in dyspareunia or severe discomfort	–	–
Vaginal discharge – A disorder characterized by vaginal secretions	Mild vaginal discharge (greater than baseline for patient)	Moderate to heavy vaginal discharge; use of perineal pad or tampon indicated	–	–	–
Vaginal inflammation - A disorder characterized by inflammation involving the vagina. Symptoms may include redness, edema, marked discomfort and an increase in vaginal discharge	Mild discomfort or pain, edema, or redness	Moderate discomfort or pain, edema, or redness; limiting instrumental ADL	Severe discomfort or pain, edema, or redness; limiting self care ADL; small areas of mucosal ulceration	Life-threatening consequences; widespread areas of mucosal ulceration; urgent intervention indicated	–
Vaginal stricture – A disorder characterized by a narrowing of the vaginal canal	Asymptomatic; mild vaginal shortening or narrowing	Vaginal narrowing and/or shortening not interfering with physical examination	Vaginal narrowing and/or shortening interfering with the use of tampons, sexual activity or physical examination	–	Death

Severe Adverse Events are defined as any of the following: any toxicity CTC Grade 4 or 5; any toxicity causing permanent or severe impairment/disability; any toxicity leading to hospitalization, malignant disease, congenital malformations/defects or any toxicity graded as SAE by the study investigator. Incidence of AEs/SAEs is assessed weekly during radiotherapy, at the end of radiotherapy as well as part of every follow-up visit. Any SAE has to be reported to the Principal Investigator within 2 days during radiotherapy and within 10 days after finishing radiotherapy, respectively. Any SAE will be documented in the electronical CRF.

#### Follow up

Patients are included into standard oncological follow-up program including regular MRI scans and colonoscopy for at least 5 years according to the current guidelines. Additionally, regular study visits at 6 weeks, 6 months and 12 months post treatment are intended. Each visit includes:

- update of medical history and documentation of the results of the latest imaging performed as part of the regular oncological follow-up
- assessment of symptoms and treatment toxicity according to the CTC AE version 5.0 criteria
- assessment of compliance regarding the regular use of the vaginal dilator
- assessment of quality of life assessed with the EORTC-QLQ30/−ANL27 questionnaires
- at 6 weeks and 12 months: measurement of the vaginal diameter using the vaginal dilator set

#### Duration of the study

Initiation of the study and inclusion of the first patient is scheduled for Q4 2019 (FPFV). Recruitment period is assumed to be 4 years to include the planned 60 patients in the study. Follow-up for each patient will be 12 months. End of study is defined as the completion of the 12 months follow-up of the last patient (LPLV), which is assumed to be in Q4 2024.

### Trial organization and coordination

The DILANA study has been designed by the study initiators at the Department of Radiation Oncology in cooperation with the Institute of Medical Biometry and Informatics at the Heidelberg University Hospital. The study is carried out by the Department of Radiation Oncology. Statistical analysis is performed by the Institute of Medical Biometry and Informatics at the University of Heidelberg. The overall coordination is performed by the Department of Radiation Oncology at University Hospital Heidelberg. This department is also responsible for the overall trial management, database management, quality assurance including monitoring and reporting.

### Investigators

The study investigators are experienced radiation oncologists specialized in the treatment of patients with gastrointestinal malignancies. Patients will be recruited and treated by the physicians of the Department of Radiation Oncology of the University Hospital Heidelberg.

### Ethics, informed consent and safety

The final protocol was approved by the ethics committee of the University of Heidelberg, Heidelberg, Germany (Nr: S-296/2019). This study complies with the Helsinki Declaration in its recent German version, the principles of Good Clinical Practice (GCP) and the Federal Data Protection Act. The trial will also be carried out in keeping with local legal and regulatory requirements. The medical secrecy and the Federal Data Protection Act will be followed. The ClinicalTrials.gov Identifier is NCT04094454.

### Data handling, storage and archiving of data

All findings including clinical and laboratory data will be documented by the investigator or an authorized member of the study team in the subject’s medical record and in the case report form (CRF). The data will be stored and archived according to the §13 of the German GCP-Regulation and §28 c of the German X-Ray Regulation (StrlSchV) for at least 30 years after the trial termination.

## Discussion

The prognosis for patients with anal cancer has improved over the last decades with complete tumor remission rates of about 80–90% today [1]. Thus, developing new therapeutic techniques in order to reduce therapy-associated long-term toxicities and to improve quality of life of anal cancer patients has become more and more important. With the development of new techniques in the field of radiation therapy like IMRT/IGRT, the incidence of acute and chronic toxicities could already be reduced [6–11]. So far, the focus has mainly been on reducing gastrointestinal toxicities. The incidence and influence of urogenital toxicities like vaginal fibrosis and stenosis on quality of life in female anal cancer patients have been widely underestimated. In the last years, only few retrospective data were published reporting on incidence, dose correlation, prevention and risk factors for vaginal fibrosis [26–30]. Furthermore, no official recommendations for prevention of vaginal stenosis exist. Current recommendations differ by center and are extrapolated from recommendations for women treated with radiotherapy for gynecological cancers. Prospective studies are needed evaluating the true incidence and extent of vaginal fibrosis, assessing possible techniques concerning radiotherapy-procedure like extended intrafractional vaginal dilation as well as other radiotherapy-independent methods like using a vaginal dilator after finishing radiotherapy to reduce the incidence of vaginal fibrosis and to evaluate the influence on quality of life in anal cancer patients. Additionally, a systematic method for assessment and measuring of vaginal stenosis should be implemented to make reported data comparable and reproducible. The aim of the current study is to assess all the mentioned aspects in a prospective setting. The systematic method for assessment of vaginal stenosis could serve as future tool for evaluating and comparing rates of vaginal stenosis. Furthermore, the clinical feasibility of the daily use of a special tampon with extended vaginal dilation will be evaluated.

## Data Availability

The datasets generated and/or analyzed during the current study are available from the corresponding author on reasonable request.

## Abbreviations

CRF: Case report form
CT: Computed tomography
CTV: Clinical Target Volume
DEGRO: German Society for Radio-oncology
ECOG: Eastern Cooperative Oncology Group
EORTC: European Organisation for Research and Treatment of Cancer
FPFV: First patient first visit
GCP: Good Clinical Practice
GTV: Gross Tumor Volume
Gy: Gray
IGRT: Image guided radiotherapy
IMRT: Intensity-modulated radiation therapy
ITT: Intention to treat
LAD: Lymphatic drainage
LPLV: Last patient last visit
NCI CTC AE v 5.0: National Cancer Institute Common Toxicity Criteria for Adverse Events version 5.0
OAR: Organ at risk
PTV: Planning Target Volume
VMAT: Volumetric Arc Therapy
